# Halloysite Nanotubes as Nano-Carriers of Corrosion Inhibitors in Cement Formulations

**DOI:** 10.3390/ma13143150

**Published:** 2020-07-15

**Authors:** Monica Tonelli, Piero Baglioni, Francesca Ridi

**Affiliations:** Department of Chemistry “Ugo Schiff” & CSGI, University of Florence, Via della Lastruccia 3 Sesto Fiorentino, I-50019 Florence, Italy; monica.tonelli@unifi.it (M.T.); piero.baglioni@unifi.it (P.B.)

**Keywords:** halloysite nanotubes, nano-silica, cementitious materials, release of active molecules, anticorrosive molecules, corrosion inhibitors, benzotriazole

## Abstract

The ingress of water, as a vehicle for many harmful substances, is the main cause of all the major physical and chemical degradation processes affecting concrete buildings. To prevent damage and protect concrete surfaces, coatings are generally used. Cement-based coatings in particular can act as a physical barrier and reduce the permeability of surfaces. In case of chloride-induced corrosion, corrosion inhibitors are also generally used, and nano-carriers have been proven to provide a long-term protective effect. In this work, we designed a surface protection cementitious coating enhanced with nano-silica and halloysite nanotubes (HNTs). HNTs were loaded with a corrosion inhibitor, benzotriazole (BTA), and used as nano-reservoir, while nano-silica was used to improve the structure of the protective coating and to strengthen its adhesion to the surface of application. The cementitious coatings were characterized with a multi-technique approach including thermal and spectroscopic analysis, scanning electron microscopy, specific surface area and pore size distribution, and Vickers hardness test. The release of BTA was monitored through UV-vis analysis, and the transportation of BTA through coated mortars was studied in simulated rain conditions. We evidenced that the presence of silica densifies the porous structure and increases the interfacial bond strength between the protective coating and the surface of application. We report here, for the first time, that HNTs can be used as nano-carriers for the slow delivery of anti-corrosion molecules in cement mortars.

## 1. Introduction

The durability of concrete structures strongly depends on their surface permeability [1,2]. When surfaces are exposed to external environmental agents, the complex network of pores and capillaries that exists in dried cementitious materials allows for the ingress of potentially dangerous substances, leading to deterioration and affecting long-term performance durability [2,3]. The ingress of water, as a vehicle for all the harmful substances, is the main cause of all the major physical and chemical degradation processes affecting concrete structures, hence the research on protective treatments of cement-based structures is extensive [3,4,5,6]. The hydrophobic treatment methods for concrete involve the decrease in the water/cement ratio (w/c ratio) [7], the use of controlled permeability formworks [8], the incorporation of water proof agents [3,4,9], and the surface treatments, including surface coating, hydrophobic impregnation, pore blocking surface treatment, and multifunctional surface treatments [3,9,10,11,12]. Among all the possibilities to enhance the service life of infrastructures, cement-based coatings can act as a physical barrier to the penetration of water, ions and gases. The preparation of a surface protection material made by cement with improved impermeability was recently reported in the literature [13]. In this article, the authors reported the study of a cement mortar enriched with nano-silica and silica fume. The use of highly reactive silica allowed the obtainment of a cement-based material with a denser structure, presenting less pores at the micrometric scale, which in turns enhances the compressive strength and impermeability. Moreover, the surface protection materials presented in the article [13] was reported to show an improved interfacial bond strength between the protective coating and the surface of application, ascribed to the refined structure of the mortar.

The corrosion of steel reinforcement by chloride ion erosion is the primary cause of weakening of structures [14]. When buildings are exposed to a marine environment or when de-icing salts are used in cold climates, chloride anions penetrating into the porous structure are frequently responsible for corrosion damage, together with atmospheric CO_2_ [4,15,16]. The chloride-induced corrosion of embedded steel in concrete is a multi-stage process: (i) ingress of the chloride-containing salts, (ii) accumulation of chlorides around reinforcement, (iii) breakdown of steel passivity, (iv) corrosion initiation and propagation [17]. Taking into account that reinforced concrete is the most widely used construction material, it becomes clear that the rapid deterioration of cement based materials due to chloride-induced corrosion involves considerable resources to repair deteriorated structures [18,19]. The prevention of reinforcement corrosion is primarily achieved by using high quality concrete and through an adequate cover. In the literature, we can find many solutions to address this problem and protect surfaces from corrosion, such as fabricating a protective coating on concrete surface [12,20], cathodic protection [21,22], conversion coating for steel [23], and adding corrosion inhibitors [24,25]. The use of corrosion inhibitors is recognized as an effective approach to prevent steel corrosion, and, despite their toxicity, traditional anticorrosive molecules are still widely used due to their exceptional efficacy against corrosion [26,27,28]. In particular, benzotriazole (BTA) and its derivatives are among the most effective corrosion inhibitors [28,29,30]. The long-term protective effect of these inhibitors can be also prolonged through a controlled release. A variety of carriers, including metal-organic framework, mixed-oxide nanoparticles, layer-by-layer assembled nanocontainers, and 2D layered materials, are used to load and release corrosion inhibitors [31,32,33]. Nanotubular clays such as halloysites (HNT) are a possible solution, being perfectly compatible with the chemical nature of the evolving phases commonly present in a cement paste [34,35], and presenting an empty tubular lumen, which can be loaded with active molecules. HNTs are aluminosilicate clay minerals (Al_2_Si_2_O_5_(OH)_4_) available in abundance in many locations around the world, they are low cost and nontoxic [36,37,38], and present high mechanical strength and modulus [39]. Nowadays, HNTs are used for many applications, involving the release of active molecules [29,33,40,41] and the increase in the mechanical performances of various materials [34,42], including Portland cement (PC) [34,43,44]. 

In this work, we designed a surface protection cementitious coating to be used forming a film on the surfaces of concrete to be protected and/or in case of delamination problems. The material here presented contains nano-silica and halloysite nanotubes loaded with a corrosion inhibitor. HNTs were loaded with BTA and used as nano-reservoir, providing a slow delivery of actives into the cementitious matrix here evidenced for the first time, opening many possibilities in the field of concrete protection. The release kinetic of BTA was monitored in simulated pore solution and in the cementitious coating, while the transportation of released BTA through coated mortars was studied in untreated samples and after simulated rain treatments. The coating was characterized through a multi-technique approach, including thermal analysis, spectroscopic analysis, scanning electron microscopy (SEM), surface area and pore size distribution investigation, and Vickers hardness test. We observed that the presence of highly active silica densifies the porous structure of the cement paste and increases the interfacial bond strength between the protective coating and the surface of application.

## 2. Materials and Methods

### 2.1. Materials

The samples were prepared with CEM I 52.5 Portland cement. Sand SATAF 113 (siliceous sand, 0.2–0.35 mm) was received by SATAF. Halloysite nanotubes (Brunauer-Emmett-Teller, BET, surface area = 26 ± 1 m^2^/g) were obtained from Imerys. Benzotriazole (99%) and fumed silica (>99%, BET surface area = 395 ± 25 m^2^/g) were supplied by Sigma Aldrich (St. Louis, MO, USA). Acetone (99.8%) was purchased from Carlo Erba (Cornaredo, Italy).

### 2.2. Loading of Halloysites

HNTs were loaded following a procedure already reported in the literature [29,40,45], slightly modified to increase the efficiency of the process. To entrap BTA, 2 g of HNTs were mixed as a dry powder with a saturated solution of BTA (4 g BTA in 50 mL of acetone). The suspension, kept under continuous stirring, was evacuated using a vacuum pump (P < 20 mbar, 3 h) and then cycled back to atmospheric pressure (1 h). Then, the process “vacuum (P < 20 mbar, 1 h)/air (atmospheric pressure, 1 h)” was repeated 2 more times, to increase the loading efficiency. Finally, HNTs were separated from solution by centrifugation, quickly washed with 10 mL of deionized water and dried (24 h, 70 °C).

### 2.3. Preparation of the Samples

Some mortar specimens were prepared to be used as surface of application, to test the efficacy of the surface protection coatings here investigated. These mortars were prepared mixing PC and sand at a sand/cement (s/c) ratio of 3, and using a water/cement (w/c) ratio of 0.5. Mortar samples were cured in cylindrical molds (r = 0.7 cm, h = 0.5 cm) at 25 °C and RH 98% for at least 28 days, then the surface was roughened with abrasive paper (P180) just before the application of the coating, to increase the contact area with the protective coating. The coatings were applied with a spatula to achieve a thickness of ≈0.5cm.

To prepare the coating formulations, powders were manually mixed and then water was incorporated and mixed with a spatula until a workable paste was obtained. We prepared mortars containing 0, 2, 4 or 8 wt% of silica, and 0 or 8 wt% of HNTs. Table 1 summarizes the composition of these coatings.

**Figure 1:**
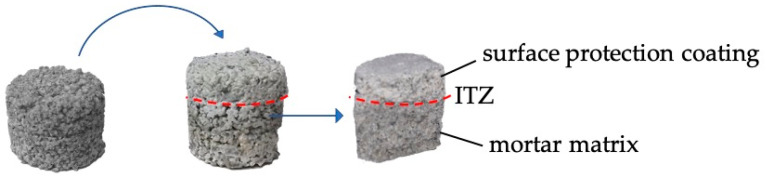
Schematic representation of a mortar before and after the application of the surface protection cementitious coatings.

After preparation, the formulations were applied onto the surface of aged standard mortars and the coated surfaces were cured at 25 °C and RH 98%. After predetermined times, the coated mortars were properly broken to study the top coating, ITZ (interfacial transition zone, interphase between the coating and the surface of application) and bottom matrix phases’ composition (see Figure 1). When necessary, specimens were lyophilized at predetermined times before the analyses. All samples were studied in triplicates.

### 2.4. Methods

#### 2.4.1. Characterization Techniques

Thermogravimetric analyses (TGA) were performed by means of a STD Q600 instrument (TA Instruments, New Castle, DE, USA), operating from room temperature to 1000 °C at 10 °C/min in nitrogen flux.

Fourier-transform infrared (FTIR) spectra were acquired with a BioRad FTS-40 spectrometer (Biorad, Cambridge, MA, USA), between 400 and 4000 cm^−1^, with a resolution of 2 cm^−1^, accumulating 32 scans. For the analysis, about 1 mg of each sample was homogenized with 100 mg of KBr and pressed to obtain a pellet.

SEM images were collected on uncoated fracture surfaces with a field-emission ΣIGMA (Carl Zeiss, Microscopy GmbH, Jena, Germany) microscope, using an accelerating potential of 5.00 kV and a working distance 7 mm. 

The surface area and pore size distribution (PSD) of the powders was measured by means of a Coulter SA 3100 analyzer (Beckman, Indianapolis, IN, USA), using nitrogen as adsorptive gas. Brunauer–Emmett–Teller [46] and Barrett–Joyne–Halenda [47] calculations were used, respectively, for the analyses of area and pore volume data.

Vickers micro-hardness tests were performed by a micro indenter (Remet HX-1000 TM, Bologna, Italy) with a Vickers tip with a load of 50 g and 10 s of indentation time. Before performing the tests, the surfaces were manually smoothed with abrasive paper (P180). To obtain reliable values, 10 points were tested for each result. The size of indentation impression was measured by an optical microscope, with the software AUTOVICKERS^®^ Remet (AUTOVICKERS software, Remet, Bologna, Italy), and all experiments were performed at room temperature.

#### 2.4.2. Kinetics of Release

The release of BTA was determined through UV-vis spectroscopy. All absorbance measurements were recorded with an Agilent Cary 3500 UV-vis spectrophotometer (Agilent, Santa Clara, CA, USA) equipped with a xenon lamp emitting in a wavelength range between 190–1100 nm. Spectra were registered at room temperature using a 500 μL quartz cells with a light path of 10 mm. The absorbance of BTA was measured at 274 nm, scanning a range between 200 and 800 nm, with 1 nm of data intervals and 0.5 s of average time.

To study the kinetic of release of BTA from HNTs through UV-vis spectroscopy, we first obtained the calibration curve of BTA in water. As a preliminary test, we investigated the release of BTA from HNTs in Simulated Pore Solution (SPS), which replicates the composition of the solution in the porosity of concrete (NaOH 5.24 g; KOH 17.94 g; CaSO_4_·2H_2_O 0.55 g; Ca(OH)_2_ 2.4 g; H_2_O 1 L). The suspension (500 mg of loaded HNTs in 50 mL SPS) was continuously stirred, aliquots were withdrawn at predetermined times and studied by UV-vis spectroscopy. Subsequently, the release of BTA from HNTs embedded in a cementitious matrix was studied. For this purpose, we investigated the release of BTA from mortars. The mortar samples were dipped in water and aliquots of the solution were withdrawn at different times, to evaluate the kinetic of the release. Taking the next step, we evaluated the migration of BTA from the top coating to the bottom of the mortar matrix in the most promising sample, S4H8. One month after the application of the coating, the specimens were broken and we extracted some pieces of the corresponding top, ITZ and bottom sections. These pieces were ground and immersed in water. After 24 h, samples were centrifuged and the solutions studied by UV-vis spectroscopy. Finally, to evaluate the possible migration of BTA from the top coating to the bottom phase in a more realistic condition, we performed some rain tests. The most promising samples were exposed to a controlled rain (moderate rain, 5 mm/h [48]) for different intervals of time (1 h rain; 3 h rain; 1 h rain repeated for 1 week; 3 h rain repeated for 1 week), then the top and bottom parts were withdrawn and the extracted pieces were dipped for 24 h into water, to evaluate the presence of BTA in the samples by looking at the UV-vis spectra of the solutions.

## 3. Results

### 3.1. Characterization of the Nanotubes

**Figure 2:**
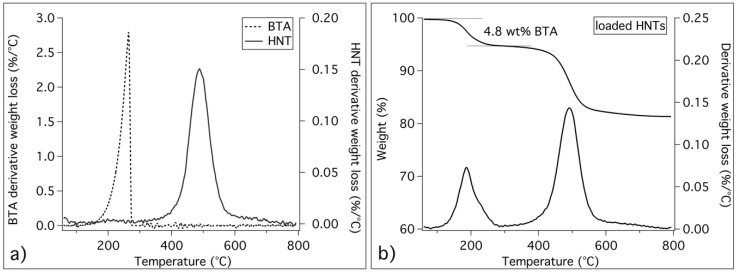
Thermogravimetric curves: (**a**) derivative thermogravimetry (DTG) curves of pristine benzotriazole (BTA) and halloysite nanotubes (HNTs); (**b**) thermogravimetry (TG)/DTG curves of loaded HNTs, together with the evaluation of BTA weight amount in loaded nanotubes.

First, HNTs and BTA were separately characterized by means of thermogravimetric analysis. Figure 2a shows that BTA decomposition starts at about 200 °C, while HNTs show a main signal centred at about 490 °C. To set the optimal conditions to entrap BTA into HNTs, we used a very concentrated BTA solution, and we monitored the pressure during the air/vacuum cycles, as explained in Section 2.2. The thermogravimetry/derivative thermogravimetry curves (TG/DTG curves) of the loaded nanotubes are shown in Figure 2b, where it is evident that BTA was successfully entrapped into HNTs. The intense signal at ≈200 °C in Figure 2b confirms that the loaded HNTs contain about 5 wt% of BTA.

### 3.2. Characterization of the Surface-Protection Cementitious Coating

#### 3.2.1. Thermogravimetric Analysis

**Figure 3:**
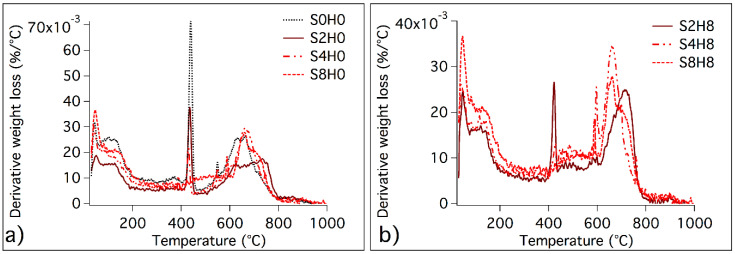
DTG curves of (**a**) S0H0, S2H0, S4H0 and S8H0; (**b**) S2H8, S4H8, S8H8 Samples were lyophilized and analysed after 28 days of hydration.

Through thermogravimetric analysis, we gained information also on the composition of the surface protection cementitious formulations after 28 days of curing, and we evaluated the effect of silica and HNTs on the hydration reactions occurring in cement pastes. Figure 3 shows the DTG curves of the investigated coatings.

The curves in Figure 3a,b allow us to discriminate the effect of silica from HNTs. The signal at about 430 °C is ascribed to portlandite (Ca(OH)_2_) and its intensity decreases with the increase in SiO_2_, due to the pozzolanic reaction that consumes portlandite in the reaction with the silica [49]. The binder gel phase calcium silicate hydrate (C–S–H), the main product of the hydration reaction, decomposes, together with aluminate phases, between room temperature and 200 °C [49,50,51], and this peak is particularly intense when SiO_2_ is present. HNTs are responsible for the peak at about 490 °C (see Figure 2b), while sand and carbonated phases are associated to the large peak between 500 and 800 °C. From the comparison between Figure 3a,b we can conclude that the presence of HNTs does not involve significant differences.

#### 3.2.2. Fourier-Transform Infrared Spectroscopy

**Figure 4:**
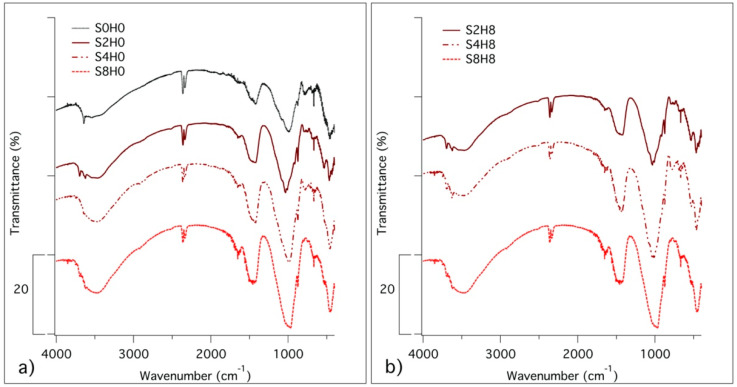
FTIR spectra of (**a**) S0H0, S2H0, S4H0 and S8H0; (**b**) S2H8, S4H8, S8H8. Samples have been lyophilized and analysed after 28 days of hydration. Spectra have been offset for the sake of clarity.

Through FTIR analysis, we further characterized the different phases present in the samples. Figure 3 shows the spectra of the same samples reported in Figure 4 for TG/DTG investigation.

Looking at the spectra in Figure 4, it is possible to confirm that SiO_2_ enhances the precipitation of C–S–H (broad peaks at 1640 and 980 cm^−1^) by favoring the consumption of Ca(OH)_2_, (signal at 3642 cm^−1^). The anhydrous phases alite and belite (peaks at about 1110 cm^−1^) are almost absent in all spectra, being the hydration reaction well advanced after one month of hydration. From the comparison of Figure 4a,b, we confirmed that the presence of HNTs (peaks at 3690, 3620 and 1115 cm^−1^) does not influence the reaction of hydration.

#### 3.2.3. Scanning Electron Microscopy of the Interfacial Transition Zone 

**Figure 5:**
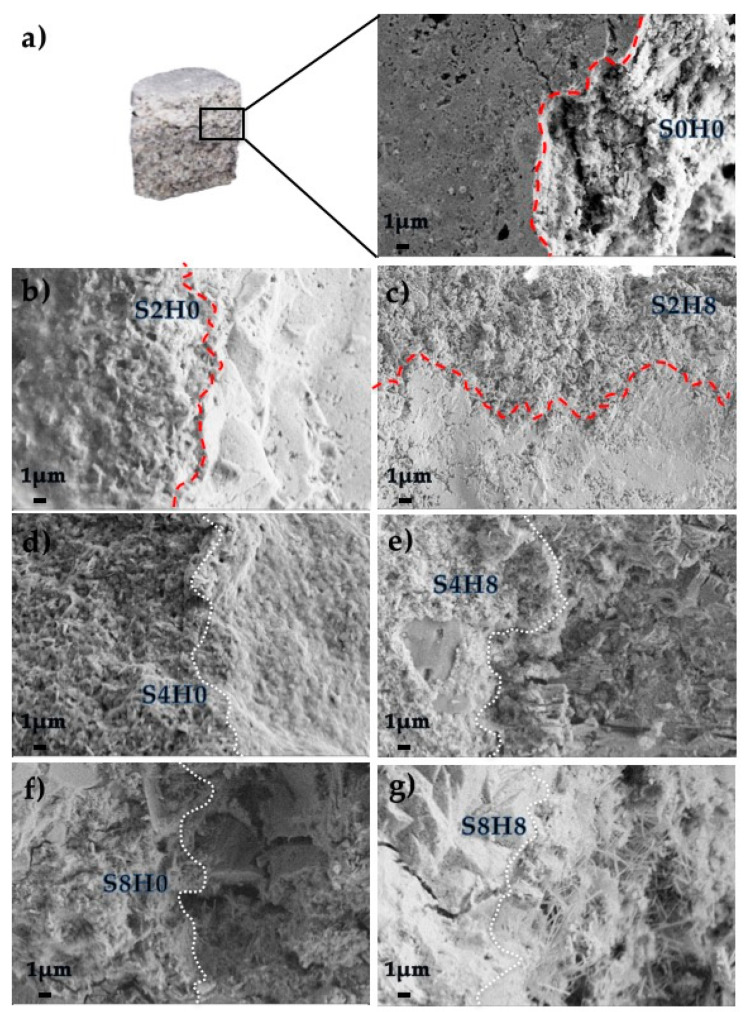
SEM images of top coating/bottom matrix interphases: (**a**) sketch of the interphase and morphology of the ITZ bottom matrix/S0H0 coating; (**b**) ITZ bottom matrix/S2H0 coating; (**c**) ITZ bottom matrix/S2H8 coating; (**d**) ITZ bottom matrix/S4H0 coating; (**e**) ITZ bottom matrix/S4H8 coating; (**f**) ITZ bottom matrix/S8H0 coating; (**g**) ITZ bottom matrix/S8H8 coating.

When breaking the samples to obtain specimens of the ITZ, we observed some differences and it was possible to qualitatively evaluate the adhesion from the rupture. For the coatings containing 0 wt% or 2 wt% of silica, it was hard to obtain specimens of the ITZ, since the top coating preferentially separated from the bottom matrix when breaking the specimens. On the other hand, the coatings containing 4 wt% or 8 wt% of silica presented a good adhesion to the bottom matrix and we could easily obtain specimens of the ITZ. The evaluation of the ITZ morphology performed by SEM (Figure 5) allows for the study of the bonding of the top coating to the aged bottom surface. The ITZ was studied both in terms of phase attachment and density differences between the top coating and the bottom. When using 0 wt% or 2 wt% of silica (S0H0, S2H0 and S2H8 samples), the ITZ can be easily recognized (see Figure 5a–c), despite the high heterogeneity of the amorphous phases in the pastes. In these conditions, the top coating can be easily distinguished from the bottom and we did not obtain a good attachment of the coating to the bottom aged samples. On the other hand, when using 4 wt% or 8 wt% of silica, we achieved a good adhesion, and it was hard to recognize the interphase (see samples S4H0, S4H8, S8H0 and S8H8 in Figure 5d–g). The coatings S4H4 and S4H8 evidenced a particularly good adhesion (Figure 5d,e), with a denser interphase, suggesting an exceptionally good top/bottom bonding.

#### 3.2.4. Specific Surface Area and Pore Size Distribution

**Figure 6:**
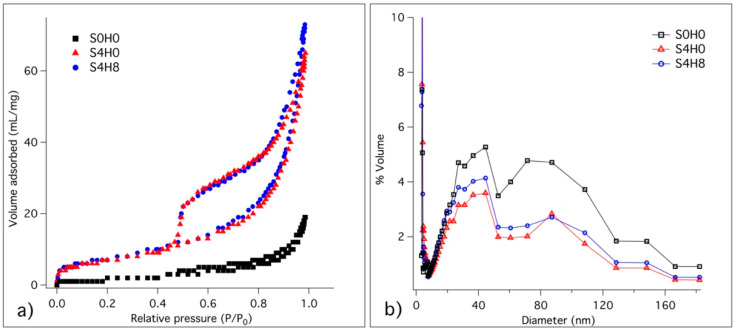
(**a**) Adsorption/desorption isotherms and (**b**) pore size distribution of S0H0, S4H0, S4H8 and S8H8. Samples have been lyophilized and analysed after 28 days of hydration.

Following the results of the previous sections, we decided to focus our attention on samples containing 4 wt% of silica, S4H0 and S4H8. The sample S0H0 was also studied for comparison. Through porosimetry, the surface area and pore size distribution of the more promising samples were measured. Figure 6 shows the adsorption/desorption isotherms (a), and the pore volume percentage distribution (b) for the investigated pastes, while Table 2 summarizes the BET surface area and total pore volume calculated from the plots.

All the physisorption isotherms (Figure 6a) are compatible with the macroporous systems of Type II, and the hysteresis loops can be classified as Type H3, which is associated with capillary condensation [52,53]. It is clear that the BET surface area of S0H0 is much lower than those of the other samples. At the same time, the distributions of pore size dimensions of the different samples (Figure 6b) are very similar, with a wide distribution of pore size. In particular, we evidenced two main populations of pores: one with an average diameter of 40 nm and another with an average diameter of 100 nm. Samples S4H0 and S4H8 present both a higher surface area and a higher pore volume with respect to S0H0 (see Table 2), due to the presence of silica, which is responsible for the enhanced formation of C–S–H.

#### 3.2.5. Vickers

**Figure 7:**
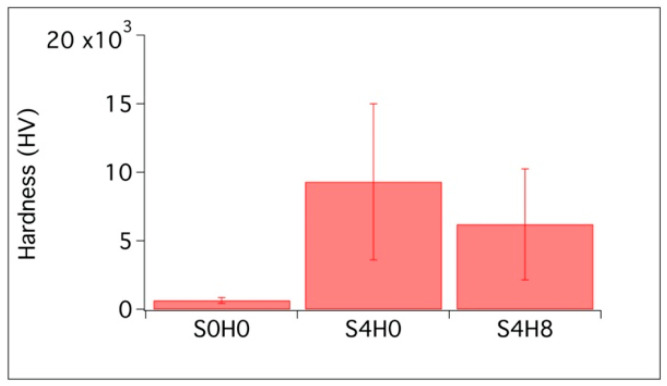
Vickers hardness test results. The average of 20 measurements is reported, together with the corresponding error bars.

The mechanical properties at the micro scale were evaluated through Vickers microindentation technique. The formulations S0H0, S4H0 and S4H8 were cured 28 days, polished and then indented with a Vickers micro-indenter (as described in Section 2.4.1). Figure 7 shows the micro-indentation results.

According to the results, the presence of fumed silica improves the mechanical properties at the micro scale, prevailing over the effect of HNTs. The hardnesses of S4H0 and S4H8 are significantly higher than S0H0 sample, with S4H0 and S4H8 showing similar mechanical properties.

### 3.3. Release of Benzotriazole in Simulated Pore Solution and in Cementitious Coating

**Figure 8:**
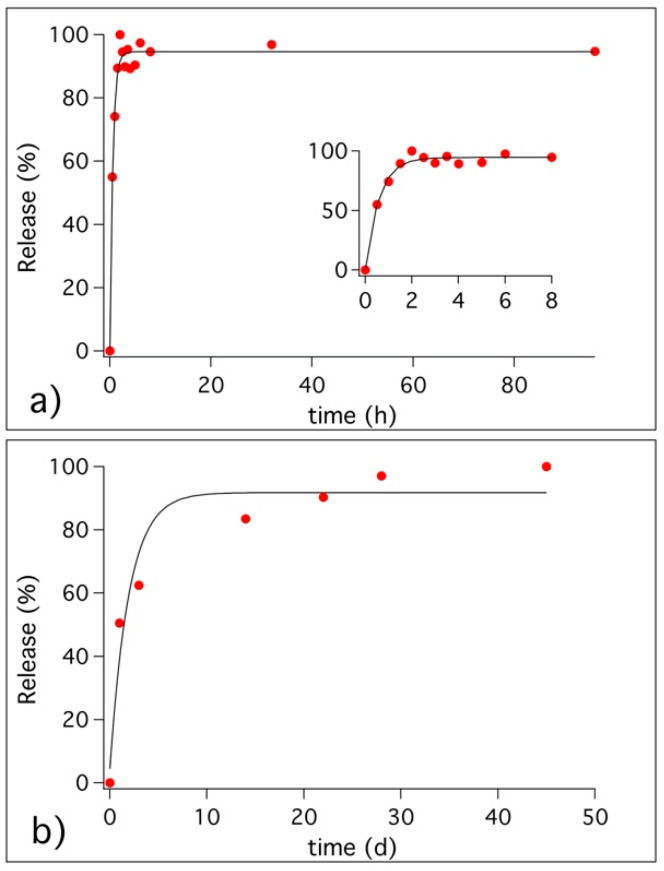
Release kinetic of BTA from HNTs: (**a**) release in simulated pore solution and zoom of the data collected in the first hours; (**b**) release in S4H8 cementitious formulation. Experimental data are reported as red markers, while black lines represent the exponential curves used to fit the collected data.

Figure 8 shows the release of BTA from HNTs in SPS (Figure 8a) and from S4H8 cementitious formulation (Figure 8b). It is important to highlight here that a BTA concentration of 5 mM was reported in the literature as successful for the protection of steel bars under chloride attack in SPS [30], which corresponds to the maximum concentration of BTA releasable from HNTs cavities when using about 2 wt% of nanotubes. To study the release kinetic in SPS, a water solution of loaded HNTs was monitored with time. We found that the release of BTA from HNTs in solution occurs in the first 90 min after the mixing, and the experimental data were fitted with an exponential curve (zoom in Figure 8a). On the other hand, to study the release from the S4H8 cementitious formulation, the mortar specimens were dipped in water one day after the preparation, and aliquots of the solution were withdrawn at different times. The maximum release of the corrosion inhibitor in these conditions (see Figure 8b) occurred in about 10 days.

### 3.4. Migration of Benzotriazole in the Cement Matrix

**Figure 9:**
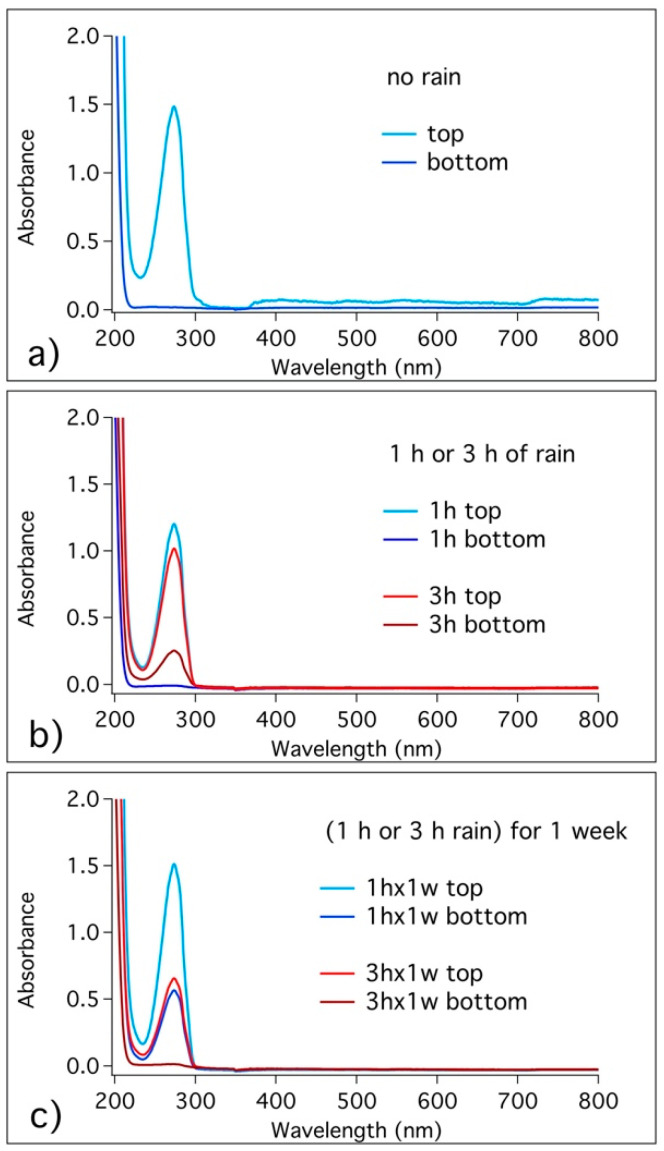
UV-vis spectra of the top and bottom piece solutions for surface coated surfaces under different conditions of rain: (**a**) no rain; (**b**) 1 h and 3 h of moderate rain; (**c**) 1 h and 3 h of moderate rain repeated for 1 week.

To evaluate the effect of rain transportation through the porosity of mortars, some mortar surfaces were coated with S4H8 formulation and cured for 7 days before examining BTA presence. It has been already evidenced that the evolution of the microstructure of cementitious matrix, together with pores size distribution, mostly occurs in the first week of hydration [12]. Coated specimens were exposed to simulated rain, then we extracted some pieces from the top coating and the bottom matrix parts and the specimens were ground and immersed 24 h in water, to assess BTA presence. For comparison, the migration of BTA from the top coating to the bottom matrix was also evaluated in absence of rain. To this purpose, some mortar matrices were coated with S4H8 and cured for one month. We decided to cure these samples for 28 days, instead of using shorter times, to allow for the migration of BTA through the cement matrix. Again, the specimens were broken to extract pieces from the top and bottom sections, and these pieces were ground and immersed in water for 24 h (other spectra were acquired also at different times of immersion in water, but no significant changes were found). Figure 9 shows the spectra of the top and bottom parts’ solutions for coated surfaces exposed to different rain conditions.

We found that BTA, released from HNTs in the top coating (see also Section 3.4), did not spontaneously migrate to the bottom part (see Figure 9a). Looking at the samples exposed to 1 h of rain (Figure 9b), again BTA was released in the top coating as expected, and we did not reveal BTA presence in the bottom matrix. Concerning the samples exposed to 3 h of rain, BTA was not only released from the top coating, but a low amount of the corrosion inhibitor was also observed in the bottom matrix. When samples were exposed to 1 h of moderate rain for one week (see Figure 9c), we observed that BTA not only was released from the top coating, but it was also transported into the bottom matrix. Finally, when samples are exposed to 3 h of rain for one week (Figure 9c), the concentration of BTA in the top coating is much lower and the bottom matrix does not reveal BTA’s presence, suggesting that the transportation through the matrix continued beyond the specimen.

## 4. Discussion

The thermal analyses performed on pristine and loaded HNTs, confirmed the successful obtainment of loaded nanotubes. The nanotubes used here contain about 5 wt% of BTA.

Data acquired by TGA and FTIR on the different coating formulations after 28 days of curing can be interpreted to get insights into the evolution of the hydration process in the presence of fumed silica and/or HNTs. The hydration of PC is a complex process in which reactants dissolve in water and hydrated phases precipitate to form a gel with a porous structure that evolves over time. In particular, calcium silicates (alite and belite) and aluminates dissolve and C–S–H binder gel forms together with portlandite and minor amounts of calcium aluminate phases [54]. According to the results, in all the formulations, we observed the expected reaction products and, by comparing the samples containing different amounts of silica, we found that in the presence of SiO_2_ the precipitation of C–S–H was enhanced. The more SiO_2_ we incorporated in the formulations, the more Ca(OH)_2_ was consumed by the pozzolanic reaction and the precipitation of the gel was enhanced. At the same time, looking at the formulations with and without HNTs, we found that the presence of the nanotubes does not involve differences in the phase composition. Thus, HNTs can be used as inorganic reinforcing additives and nano-carriers into cementitious coatings without affecting the hydrated phases precipitating in the paste.

The morphologies of the interphases of surface coated matrices were investigated by SEM. The ITZ was studied both in terms of phase attachment and density differences between the top coating and the bottom. When using 0 wt% or 2 wt% of silica the ITZ could be easily recognized and the specimens preferentially broke at the interphase, while in samples containing 4 wt% or 8 wt% of silica, we achieved a good adhesion and the morphology of the interphase was hardly recognizable, given that the coating bonded well with the surface of application. As already reported in the literature for similar formulations, the presence of nano-silica refines the ITZ, enhancing the formation of C–S–H, and leading to a denser pore structure and improved interfacial bond strength [13]. In this work, the formulations containing 4 wt% of silica (S4H0 and S4H8) evidenced the best performances and were selected for the next analyses.

Through porosimetry, we further confirmed that the presence of silica enhances the formation of hydrated phases and densify the structure, which in turns enhances the compressive strength and impermeability of cement formulations [13]. When adding 4 wt% of SiO_2_, the BET surface area and total pore volume increased and this effect result from to the improved formation of hydrated phases. In cement paste, the microstructure evolves through the progressive closure of porosity, called depercolation, which, in turn, affects the transportation properties that, in most degradation mechanisms, govern the rate of damage [55]. The progressive formation of hydrated phases and smaller pores produces an increase in the surface area and of the total pore volume [55], as observed in S4H0 and S4H8 samples.

In the literature, calcined kaolinitic clays exhibited considerable influence in enhancing the mechanical properties and the durability of mortar and concrete. Furthermore, it has been reported that, for tubular halloysite, the reaction of alumina occurs later than in other kaolinitic clays, and the production of different assemblages of hydrated phases contributes to enhance the compressive strength at later stages [56]. In this work, we evaluated the hardness at the micro scale through Vickers micro-indentation and we found that that silica has a major effect on the enhancement of the mechanical properties. The hardness at the microscale is mostly influenced by the SiO_2_ presence, which improves the mechanical properties through the formation of a denser porous cementitious structure. On the other hand, we did not detect a significant reinforcement connected to HNTs, probably due to the strong heterogeneity of the samples when observed at the microscale.

Taking the next step, we evaluated the efficiency of HNTs as nano-carriers. The release of BTA corrosion inhibitor from HNTs dispersed in SPS occurred in about 90 min, while it took weeks for BTA to be completely released when loaded HNTs were incorporated in cement mortars. Hence, we found that BTA can be released not only in solution, but also in cementitious matrix. Nevertheless, the released BTA did not spontaneously migrate through the mortars. To achieve the migration of BTA through the porosity of the mortars, water transportation was necessary, occurring as a result of rain events. When samples were exposed to simulated rain, BTA was carried from the top coating, where it was released from HNT cavities to the bottom matrix and beyond. Samples exposed to 1h of moderate rain (5 mm/h) per day for 1 week resulted in a BTA migration of about 1 cm (the thickness of the investigated coated specimen), while samples exposed to 1 h of rain per day for 1 week result in a BTA migration higher than 1 cm. In the latter case, the transportation of BTA into the cementitious matrix occurred so effectively that a consistent amount of anticorrosive passed through the samples and went out. The results suggest that it is possible to modulate the release of BTA from HNTs incorporated into cementitious surface coatings. We found that the release and transportation of BTA occurs when water penetrates into the porous matrix, and the corrosion inhibitor can be transported into the mortar to accomplish its protecting function.

## 5. Conclusions

This study explored the effects of HNTs and nano-silica in cement mortars. We designed a surface protection cementitious coating containing HNTs loaded with corrosion inhibitors and silica, for the preservation of dried cementitious materials. Based on the results, we achieved an adequate loading of BTA into the cavities of HNTs and we evidenced that it is possible to release the corrosion inhibitor not only in solution, but also in cement mortars. The presence of nano-silica allowed us to obtain a good adhesion between the coating and the surface of application, densifying the porous structure at the interphase and enhancing the precipitation of C–S–H binder gel. In particular, the use of 4 wt% of silica was found to be the best amount.

The ingress of water in cementitious materials is generally considered a vehicle for all the harmful substances. Here, we found that, in the case of water transportation, we can also achieve the release and transportation of the BTA corrosion inhibitor through the cement matrix. Thus, the protection coating here presented is particularly effective and we demonstrated that BTA, released from the nano-carriers, can migrate with water to reach the internal matrix for the prevention of corrosion degradation mechanisms.

## Figures and Tables

**Table 1:** Composition of the cementitious coatings.

Name	SiO_2_ (wt%) ^1^	HNT (wt%) ^1^	s/c	w/c
S0H0	0	0	3	0.5
S2H0	2	0	3	1.2
S2H8	2	8	3	1.2
S4H0	4	0	3	1.8
S4H8	4	8	3	1.8
S8H0	8	0	3	2.4
S4H8	8	8	3	2.4

^1^ weight percentages are calculated with respect to the total weight of cement + sand.

**Table 2:** BET surface area and total pore volume of S0H0, S4H0 and S4H8 samples.

Sample	BET Surface Area (m^2^/g)	Total Pore Volume (mL/g)
S0H0	7 ± 1	0.03 ± 0.01
S4H0	27 ± 1	0.10 ± 0.01
S4H8	29 ± 1	0.11 ± 0.01
